# Crystal structure of 1-benzyl-4-(4-chloro­phen­yl)-2-imino-1,2,5,6,7,8,9,10-octa­hydro­cyclo­octa­[*b*]pyridine-3-carbo­nitrile

**DOI:** 10.1107/S160053681401962X

**Published:** 2014-09-06

**Authors:** R. A. Nagalakshmi, J. Suresh, S. Maharani, R. Ranjith Kumar, P. L. Nilantha Lakshman

**Affiliations:** a Department of Physics, The Madura College, Madurai 625 011, India; b Department of Organic Chemistry, School of Chemistry, Madurai Kamaraj University, Madurai 625 021, India; c Department of Food Science and Technology, University of Ruhuna, Mapalana, Kamburupitiya 81100, Sri Lanka

**Keywords:** crystal structure, cyclo­octa­[*b*]pyridine, hydrogen bonding, Schiff bases

## Abstract

The title compound, C_25_H_24_ClN_3_, comprises a 2-imino­pyridine ring fused with a cyclo­octane ring, which adopts a twist boat–chair conformation. In the crystal, C—H⋯N inter­actions form *R*_2_^2^(14) ring motifs and mol­ecules are further connected by weak C—H⋯π inter­actions. The resulting supra­molecular structure is a two-dimensional framework parallel to the *ab* plane.

## Abstract 2

The title compound comprises a 2-imino­pyridine ring fused with a cyclo­octane ring, which adopts a twist boat–chair conformation. Inter­molecular C—H⋯N inter­actions form 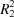
(14) ring motifs and mol­ecules are further connected by weak C—H⋯π inter­actions.

## Chemical context   

Schiff bases are compounds carrying an imine or azomethine (—C=N—) functional group. They have gained importance in the medicinal and pharmaceutical fields due to their broad spectrum of biological activity, including anti-inflammatory, analgesic, anti­microbial, anti­convulsant, anti­tubercular (Aboul-Fadl *et al.*, 2003 ▶), anti­cancer, anti­oxidant and anti­helminthic, among others. Schiff base derivatives are present in a number of processes, which prompted researchers to design novel heterocyclic/aryl Schiff bases with the aim of developing new environmentally friendly technologies (Bhattacharya *et al.*, 2003 ▶). Schiff bases are also used as ligands for catalysts, inter­mediates in organic synthesis, dyes, pigments, and polymer stabilizers (Dhar & Taploo, 1982 ▶).
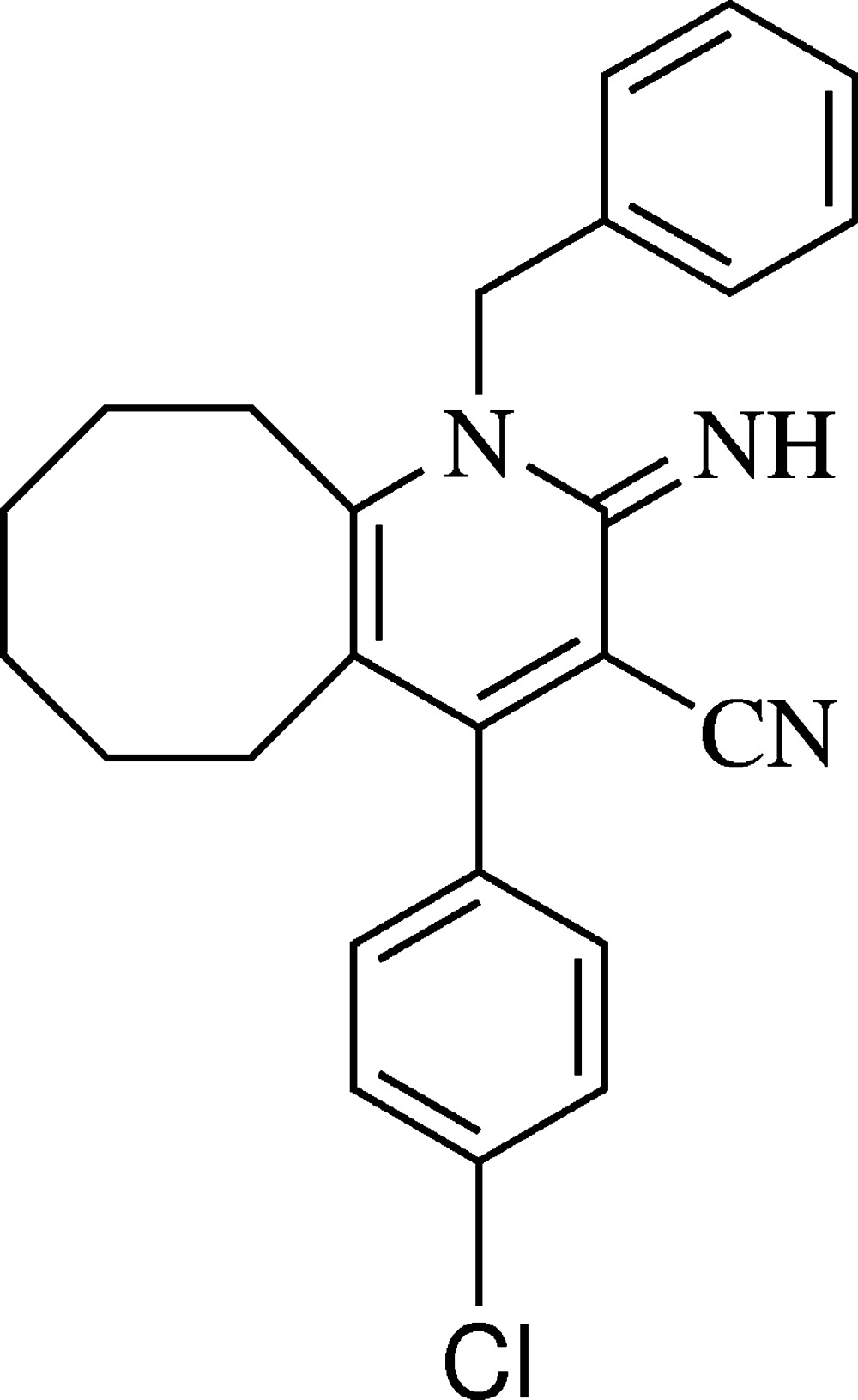


Imino­pyridine complexes can be useful catalysts, and pyridones have been investigated extensively as valuable building blocks for many fused heterocyclic systems (Johns *et al.*, 2003 ▶) displaying a wide range of biological and pharmacological activities. They exhibit, for example, anti­proliferative and anti­tubolin activities (Magedov *et al.*, 2008 ▶). Many pyridin-2-one and 3-cyano-2-imino­pyridine derivatives also exhibit anti­proliferative activity (McNamara & Cook, 1987 ▶). As part of our studies in this area, the title compound was synthesized and we report herein on the mol­ecular and crystal structures of this compound.

## Structural commentary   

**Figure 1:**
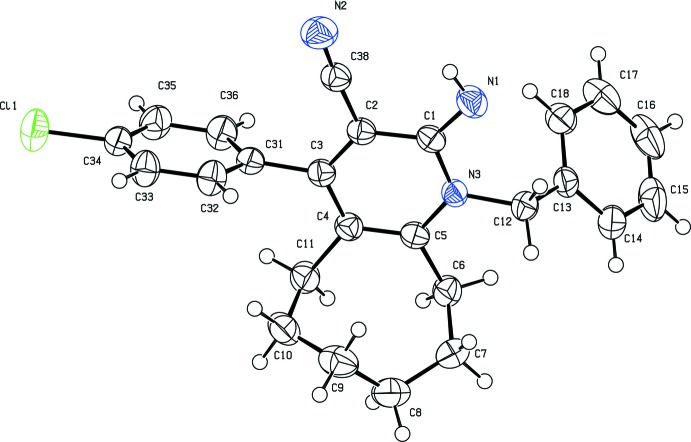
The mol­ecular structure of the title compound, showing 20% probability displacement ellipsoids. All H atoms have been omitted for clarity.

The mol­ecular structure of the title compound is shown in Fig. 1 ▶. The cyclo­octane ring adopts a twist boat–chair conformation (Wiberg, 2003 ▶), as found in similar structures (Vishnupriya *et al.*, 2014*a* ▶,*b* ▶). As expected, the pyridine ring (atoms C1–C5/N3) is almost planar, with an r.m.s. deviation of 0.002 Å. The chloro­benzene (C31–C36) and phenyl (C13–C18) rings are almost planar, with r.m.s. deviations of 0.005 and 0.004 Å, respectively. The sum of the angles around atom N3 is 359.8°, indicating that atom N3 is *sp* ^2^-hybridized. The C2—C38 N2 bond angle of 176.07 (19)° shows the linearity of the cyano group, a feature systematically observed in carbo­nitrile compounds. Nitrile atoms C38 and N2 are displaced from the mean plane of the pyridine ring by 0.0258 (1) and 0.0363 (1) Å, respectively. The imino C1=N1 bond length is 1.286 (2) Å. The imino group is nearly coplanar with the pyridine ring, as indicated by the N1=C1—N3—C5 torsion angle of −178.89 (14)°. The chloro­benzene ring is attached to the pyridine ring with a C2=C3—C31 C36 torsion angle of 100.99 (19)°, indicating a (+)anti­clinal conformation. The C33 C34 C35 bond angle of 121.11 (15)° deviates from 120° due to the presence of the chlorine substituent. The chlorine atom bonded to C34 deviates by 0.0446 (1) Å from the mean plane of the phenyl ring. The chlorine is attached to the benzene ring with a C32 C33 C34—Cl1 torsion angle of 178.95 (13)°. In the pyridine ring, the formal double bonds [C4=C5 = 1.375 (2) and C2=C3 = 1.369 (2) Å] are longer than standard C=C bonds (1.34 Å), while the other bond lengths are slightly shorter than standard C—C and C—N bond lengths, evidencing that there is a homo-conjugation effect for this ring.

## Supra­molecular features   

**Figure 2:**
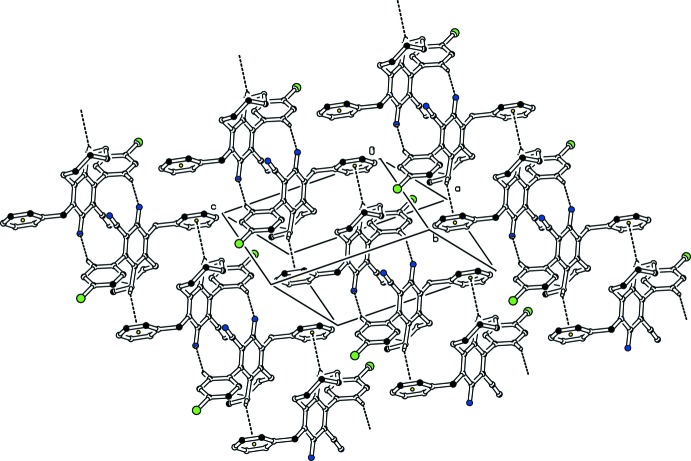
Partial packing diagram of the title compound. Dashed lines represent inter­molecular hydrogen bonds and C—H⋯π contacts. For clarity, H atoms not involved in hydrogen bonding have been omitted.

In the crystal, pairs of C—H⋯N inter­actions form 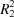
(14) ring motifs (Bernstein *et al.*, 1995 ▶), and the resulting dimers are further connected through weak C—H⋯π inter­actions involving the phenyl ring as acceptor (Table 1 ▶ and Fig. 2 ▶). The resulting supra­molecular structure is a two-dimensional framework parallel to the crystallographic *ab* plane.

## Database survey   

Similar structures reported in the literature are 2-meth­oxy-4-(2-meth­oxy­phen­yl)-5,6,7,8,9,10-hexa­hydro­cyclo­octa­[*b*]pyrid­ine-3-carbo­nitrile (Vishnupriya *et al.*, 2014*a* ▶) and 4-(2-fluoro­phen­yl)-2-meth­oxy-5,6,7,8,9,10-hexa­hydro­cyclo­octa­[*b*]pyridine-3-carbo­nitrile (Vishnupriya *et al.*, 2014*b* ▶). In the structure reported here, the twisted conformation of the cyclo­octane ring and the planar conformation of the pyridine are similar to those found in the related structures. However, the C=NH functional group present in the title compound allows the formation of C—H⋯N hydrogen bonds, which are not present in the above-cited compounds. In the title compound, the bond lengths in the central pyridine ring span the range 1.369–1.447 Å, which compares well with the ranges observed in the similar structures (1.314–1.400 Å), but these bonds are systematically longer in the title compound, due to the substitution of the pyridine N atom by a benzyl group.

## Synthesis and crystallization   

Cyclo­octa­none (1 mmol), 4-chloro­benzaldehyde (1 mmol) and malono­nitrile (1 mmol) were mixed in ethanol (10 ml), and *p*-toluene­sulfonic acid (0.5 mmol) was added. The reaction mixture was refluxed for 2–3 h. After completion of the reaction (followed by thin-layer chromatography), the mixture was poured into crushed ice and extracted with ethyl acetate. The excess of solvent was removed under reduced pressure and the residue was chromatographed using a petroleum ether/ethyl acetate mixture (97:3 *v*/*v*) as eluent, to afford the pure product. The product was recrystallized from ethyl acetate, affording colourless crystals (m.p. 493 K; yield 71%).

## Refinement   

C-bound H atoms were placed in calculated positions and allowed to ride on their carrier atoms, with C—H = 0.93 (aromatic CH) or 0.97 Å (methyl­ene CH_2_). Imine atom H1 was found in a difference map and refined freely, with the N—H distance restrained to 0.84 (2) Å. Isotropic displacement parameters for H atoms were calculated as *U*_iso_(H) = 1.2*U*_eq_(C) for CH and CH_2_ groups, while the *U*_iso_ factor for H1 was refined. Crystal data, data collection and structure refinement details are summarized in Table 2. ▶

## Supplementary Material

Crystal structure: contains datablock(s) global, I. DOI: 10.1107/S160053681401962X/bh2503sup1.cif

Structure factors: contains datablock(s) I. DOI: 10.1107/S160053681401962X/bh2503Isup2.hkl

CCDC reference: 1021949

Additional supporting information:  crystallographic information; 3D view; checkCIF report

## Figures and Tables

**Table 1:** Hydrogen-bond geometry (Å, °) *Cg*1 is the centroid of the phenyl ring.

*D*—H⋯*A*	*D*—H	H⋯*A*	*D*⋯*A*	*D*—H⋯*A*
C32—H32⋯N1^i^	0.93	2.55	3.423 (2)	156
C11—H11*B*⋯*Cg*1^ii^	0.97	2.91	3.5642 (2)	126

Symmetry codes: (i) 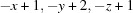; (ii) 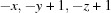.

**Table 2:** Experimental details

Crystal data	
Chemical formula	C_25_H_24_ClN_3_
*M*_r_	401.92
Crystal system, space group	Triclinic, *P* 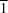
Temperature (K)	293
*a*, *b*, *c* (Å)	10.2319 (3), 10.5228 (3), 11.7767 (4)
α, β, γ (°)	101.088 (2), 107.524 (2), 114.008 (2)
*V* (Å^3^)	1029.87 (5)
*Z*	2
Radiation type	Mo *K*α
μ (mm^−1^)	0.20
Crystal size (mm)	0.21 × 0.19 × 0.18

Data collection	
Diffractometer	Bruker Kappa APEXII
Absorption correction	Multi-scan (*SADABS*; Bruker, 2004 ▶)
*T*_min_, *T*_max_	0.967, 0.974
No. of measured, independent and observed [*I* > 2σ(*I*)] reflections	26728, 3842, 3094
*R*_int_	0.027
(sin θ/λ)_max_ (Å^−1^)	0.606

Refinement	
*R*[*F* ^2^ > 2σ(*F* ^2^)], *wR*(*F* ^2^), *S*	0.038, 0.105, 1.05
No. of reflections	3842
No. of parameters	266
No. of restraints	2
H-atom treatment	H atoms treated by a mixture of independent and constrained refinement
Δρ_max_, Δρ_min_ (e Å^−3^)	0.29, −0.33

Computer programs: *APEX2* and *SAINT* (Bruker, 2004 ▶), *SHELXS97* and *SHELXL97* (Sheldrick, 2008 ▶) and *PLATON* (Spek, 2009 ▶).

## supplementary crystallographic information

### Crystal data

**Table:**

C_25_H_24_ClN_3_	*Z* = 2
*M**_r_* = 401.92	*F*(000) = 424
Triclinic, *P*1	*D*_x_ = 1.296 Mg m^−^^3^
Hall symbol: -P 1	Melting point: 493 K
*a* = 10.2319 (3) Å	Mo *K*α radiation, λ = 0.71073 Å
*b* = 10.5228 (3) Å	Cell parameters from 2000 reflections
*c* = 11.7767 (4) Å	θ = 2–31°
α = 101.088 (2)°	µ = 0.20 mm^−^^1^
β = 107.524 (2)°	*T* = 293 K
γ = 114.008 (2)°	Block, colourless
*V* = 1029.87 (5) Å^3^	0.21 × 0.19 × 0.18 mm

### Data collection

**Table:**

Bruker Kappa APEXII diffractometer	3842 independent reflections
Radiation source: fine-focus sealed tube	3094 reflections with *I* > 2σ(*I*)
Graphite monochromator	*R*_int_ = 0.027
Detector resolution: 0 pixels mm^-1^	θ_max_ = 25.5°, θ_min_ = 2.3°
ω and φ scans	*h* = −12→12
Absorption correction: multi-scan (*SADABS*; Bruker, 2004)	*k* = −12→12
*T*_min_ = 0.967, *T*_max_ = 0.974	*l* = −14→14
26728 measured reflections	

### Refinement

**Table:**

Refinement on *F*^2^	Primary atom site location: structure-invariant direct methods
Least-squares matrix: full	Secondary atom site location: difference Fourier map
*R*[*F*^2^ > 2σ(*F*^2^)] = 0.038	Hydrogen site location: inferred from neighbouring sites
*wR*(*F*^2^) = 0.105	H atoms treated by a mixture of independent and constrained refinement
*S* = 1.05	*w* = 1/[σ^2^(*F*_o_^2^) + (0.0436*P*)^2^ + 0.4103*P*] where *P* = (*F*_o_^2^ + 2*F*_c_^2^)/3
3842 reflections	(Δ/σ)_max_ < 0.001
266 parameters	Δρ_max_ = 0.29 e Å^−^^3^
2 restraints	Δρ_min_ = −0.33 e Å^−^^3^
0 constraints	

### Fractional atomic coordinates and isotropic or equivalent isotropic displacement parameters (Å^2^)

**Table:**

	*x*	*y*	*z*	*U*_iso_*/*U*_eq_	
C1	0.41432 (18)	0.89760 (17)	0.60601 (14)	0.0337 (3)	
C2	0.43550 (18)	0.80064 (17)	0.51697 (14)	0.0335 (3)	
C3	0.31232 (18)	0.67411 (17)	0.41791 (14)	0.0333 (3)	
C4	0.15512 (18)	0.63430 (17)	0.39917 (14)	0.0353 (3)	
C5	0.13131 (18)	0.72425 (17)	0.48236 (14)	0.0335 (3)	
C6	−0.03192 (19)	0.6888 (2)	0.46876 (16)	0.0423 (4)	
H6A	−0.0265	0.7273	0.5528	0.051*	
H6B	−0.0988	0.5812	0.4361	0.051*	
C7	−0.1099 (2)	0.7521 (2)	0.38092 (18)	0.0551 (5)	
H7A	−0.1975	0.7499	0.3983	0.066*	
H7B	−0.0338	0.8559	0.4038	0.066*	
C8	−0.1713 (2)	0.6742 (3)	0.23817 (19)	0.0593 (5)	
H8A	−0.2427	0.5691	0.2160	0.071*	
H8B	−0.2325	0.7141	0.1940	0.071*	
C9	−0.0483 (2)	0.6871 (2)	0.18839 (19)	0.0578 (5)	
H9A	0.0513	0.7749	0.2484	0.069*	
H9B	−0.0788	0.7029	0.1083	0.069*	
C10	−0.0220 (2)	0.5534 (2)	0.16653 (17)	0.0557 (5)	
H10A	0.0628	0.5763	0.1392	0.067*	
H10B	−0.1169	0.4690	0.0970	0.067*	
C11	0.0186 (2)	0.50636 (19)	0.28133 (17)	0.0456 (4)	
H11A	−0.0733	0.4650	0.2993	0.055*	
H11B	0.0448	0.4287	0.2603	0.055*	
C12	0.2287 (2)	0.95489 (18)	0.66307 (15)	0.0397 (4)	
H12A	0.1371	0.9574	0.6102	0.048*	
H12B	0.3189	1.0545	0.6960	0.048*	
C13	0.20272 (19)	0.91624 (18)	0.77398 (15)	0.0386 (4)	
C14	0.0786 (2)	0.9164 (2)	0.79756 (18)	0.0550 (5)	
H14	0.0085	0.9357	0.7426	0.066*	
C15	0.0579 (3)	0.8877 (3)	0.9030 (2)	0.0687 (7)	
H15	−0.0265	0.8871	0.9179	0.082*	
C16	0.1608 (3)	0.8603 (2)	0.98475 (19)	0.0672 (6)	
H16	0.1475	0.8427	1.0560	0.081*	
C17	0.2835 (2)	0.8589 (2)	0.96164 (18)	0.0570 (5)	
H17	0.3531	0.8395	1.0171	0.068*	
C18	0.3047 (2)	0.88603 (19)	0.85687 (16)	0.0444 (4)	
H18	0.3881	0.8841	0.8417	0.053*	
C31	0.34478 (18)	0.57918 (17)	0.33174 (15)	0.0352 (3)	
C32	0.3811 (2)	0.61741 (19)	0.23496 (17)	0.0434 (4)	
H32	0.3887	0.7054	0.2250	0.052*	
C33	0.4064 (2)	0.5266 (2)	0.15252 (17)	0.0458 (4)	
H33	0.4291	0.5523	0.0868	0.055*	
C34	0.39752 (19)	0.39863 (18)	0.16899 (15)	0.0394 (4)	
C35	0.3645 (2)	0.3594 (2)	0.26548 (18)	0.0488 (4)	
H35	0.3603	0.2728	0.2764	0.059*	
C36	0.3376 (2)	0.4497 (2)	0.34654 (17)	0.0472 (4)	
H36	0.3143	0.4229	0.4118	0.057*	
C38	0.59554 (19)	0.84653 (18)	0.53780 (15)	0.0384 (4)	
N1	0.52304 (18)	1.01684 (16)	0.70223 (14)	0.0466 (4)	
N2	0.72609 (18)	0.88953 (19)	0.56079 (16)	0.0557 (4)	
N3	0.25617 (15)	0.85167 (14)	0.58174 (11)	0.0328 (3)	
Cl1	0.42569 (7)	0.28161 (6)	0.06488 (5)	0.06290 (17)	
H1	0.612 (2)	1.031 (2)	0.703 (2)	0.063 (6)*	

### Atomic displacement parameters (Å^2^)

**Table:**

	*U*^11^	*U*^22^	*U*^33^	*U*^12^	*U*^13^	*U*^23^
C1	0.0364 (8)	0.0339 (8)	0.0297 (8)	0.0161 (7)	0.0126 (6)	0.0147 (7)
C2	0.0346 (7)	0.0360 (8)	0.0313 (8)	0.0174 (7)	0.0139 (6)	0.0156 (6)
C3	0.0372 (8)	0.0341 (8)	0.0315 (8)	0.0188 (7)	0.0147 (7)	0.0148 (7)
C4	0.0340 (8)	0.0334 (8)	0.0342 (8)	0.0153 (7)	0.0123 (7)	0.0103 (7)
C5	0.0348 (8)	0.0348 (8)	0.0318 (8)	0.0170 (7)	0.0133 (6)	0.0154 (7)
C6	0.0387 (9)	0.0476 (10)	0.0403 (9)	0.0205 (8)	0.0198 (7)	0.0125 (8)
C7	0.0475 (10)	0.0707 (13)	0.0534 (11)	0.0388 (10)	0.0184 (9)	0.0181 (10)
C8	0.0463 (11)	0.0748 (14)	0.0540 (12)	0.0336 (10)	0.0132 (9)	0.0229 (10)
C9	0.0490 (11)	0.0732 (14)	0.0458 (10)	0.0252 (10)	0.0167 (9)	0.0278 (10)
C10	0.0427 (10)	0.0670 (13)	0.0362 (9)	0.0196 (9)	0.0110 (8)	0.0034 (9)
C11	0.0360 (9)	0.0398 (9)	0.0466 (10)	0.0141 (7)	0.0141 (8)	0.0030 (8)
C12	0.0460 (9)	0.0373 (9)	0.0376 (9)	0.0256 (8)	0.0151 (7)	0.0101 (7)
C13	0.0387 (8)	0.0359 (8)	0.0334 (8)	0.0168 (7)	0.0141 (7)	0.0028 (7)
C14	0.0465 (10)	0.0593 (12)	0.0484 (11)	0.0274 (9)	0.0172 (9)	0.0002 (9)
C15	0.0530 (12)	0.0728 (14)	0.0585 (13)	0.0171 (11)	0.0346 (11)	−0.0048 (11)
C16	0.0625 (13)	0.0677 (14)	0.0382 (10)	0.0069 (11)	0.0264 (10)	0.0034 (10)
C17	0.0534 (11)	0.0595 (12)	0.0379 (10)	0.0146 (9)	0.0149 (9)	0.0155 (9)
C18	0.0399 (9)	0.0479 (10)	0.0389 (9)	0.0179 (8)	0.0162 (7)	0.0127 (8)
C31	0.0320 (8)	0.0351 (8)	0.0345 (8)	0.0160 (7)	0.0117 (7)	0.0099 (7)
C32	0.0541 (10)	0.0374 (9)	0.0494 (10)	0.0248 (8)	0.0291 (8)	0.0203 (8)
C33	0.0572 (11)	0.0464 (10)	0.0460 (10)	0.0272 (9)	0.0319 (9)	0.0211 (8)
C34	0.0371 (8)	0.0394 (9)	0.0397 (9)	0.0203 (7)	0.0152 (7)	0.0090 (7)
C35	0.0627 (11)	0.0458 (10)	0.0535 (11)	0.0357 (9)	0.0273 (9)	0.0245 (9)
C36	0.0631 (11)	0.0526 (11)	0.0452 (10)	0.0362 (9)	0.0304 (9)	0.0270 (8)
C38	0.0372 (8)	0.0421 (9)	0.0357 (8)	0.0188 (7)	0.0153 (7)	0.0162 (7)
N1	0.0411 (8)	0.0414 (8)	0.0408 (8)	0.0139 (7)	0.0126 (7)	0.0049 (7)
N2	0.0407 (9)	0.0659 (11)	0.0566 (10)	0.0236 (8)	0.0201 (7)	0.0214 (8)
N3	0.0374 (7)	0.0333 (7)	0.0289 (6)	0.0190 (6)	0.0136 (5)	0.0112 (5)
Cl1	0.0826 (4)	0.0577 (3)	0.0635 (3)	0.0439 (3)	0.0404 (3)	0.0156 (2)

### Geometric parameters (Å, º)

**Table:**

C1—N1	1.286 (2)	C12—N3	1.4786 (19)
C1—N3	1.402 (2)	C12—C13	1.506 (2)
C1—C2	1.447 (2)	C12—H12A	0.9700
C2—C3	1.369 (2)	C12—H12B	0.9700
C2—C38	1.430 (2)	C13—C14	1.380 (2)
C3—C4	1.419 (2)	C13—C18	1.385 (2)
C3—C31	1.490 (2)	C14—C15	1.388 (3)
C4—C5	1.375 (2)	C14—H14	0.9300
C4—C11	1.508 (2)	C15—C16	1.365 (3)
C5—N3	1.379 (2)	C15—H15	0.9300
C5—C6	1.504 (2)	C16—C17	1.368 (3)
C6—C7	1.533 (3)	C16—H16	0.9300
C6—H6A	0.9700	C17—C18	1.377 (2)
C6—H6B	0.9700	C17—H17	0.9300
C7—C8	1.519 (3)	C18—H18	0.9300
C7—H7A	0.9700	C31—C32	1.382 (2)
C7—H7B	0.9700	C31—C36	1.382 (2)
C8—C9	1.510 (3)	C32—C33	1.385 (2)
C8—H8A	0.9700	C32—H32	0.9300
C8—H8B	0.9700	C33—C34	1.367 (2)
C9—C10	1.527 (3)	C33—H33	0.9300
C9—H9A	0.9700	C34—C35	1.369 (2)
C9—H9B	0.9700	C34—Cl1	1.7387 (16)
C10—C11	1.527 (3)	C35—C36	1.383 (2)
C10—H10A	0.9700	C35—H35	0.9300
C10—H10B	0.9700	C36—H36	0.9300
C11—H11A	0.9700	C38—N2	1.143 (2)
C11—H11B	0.9700	N1—H1	0.861 (15)
			
N1—C1—N3	118.60 (15)	C10—C11—H11B	109.1
N1—C1—C2	127.15 (15)	H11A—C11—H11B	107.8
N3—C1—C2	114.24 (13)	N3—C12—C13	115.09 (13)
C3—C2—C38	121.37 (14)	N3—C12—H12A	108.5
C3—C2—C1	123.18 (14)	C13—C12—H12A	108.5
C38—C2—C1	115.45 (14)	N3—C12—H12B	108.5
C2—C3—C4	119.51 (14)	C13—C12—H12B	108.5
C2—C3—C31	119.70 (14)	H12A—C12—H12B	107.5
C4—C3—C31	120.79 (13)	C14—C13—C18	118.66 (17)
C5—C4—C3	118.62 (14)	C14—C13—C12	119.81 (16)
C5—C4—C11	121.18 (14)	C18—C13—C12	121.48 (15)
C3—C4—C11	119.80 (14)	C13—C14—C15	120.3 (2)
C4—C5—N3	121.43 (14)	C13—C14—H14	119.9
C4—C5—C6	121.59 (14)	C15—C14—H14	119.9
N3—C5—C6	116.98 (13)	C16—C15—C14	120.32 (19)
C5—C6—C7	114.83 (14)	C16—C15—H15	119.8
C5—C6—H6A	108.6	C14—C15—H15	119.8
C7—C6—H6A	108.6	C15—C16—C17	119.81 (19)
C5—C6—H6B	108.6	C15—C16—H16	120.1
C7—C6—H6B	108.6	C17—C16—H16	120.1
H6A—C6—H6B	107.5	C16—C17—C18	120.5 (2)
C8—C7—C6	116.81 (16)	C16—C17—H17	119.8
C8—C7—H7A	108.1	C18—C17—H17	119.8
C6—C7—H7A	108.1	C17—C18—C13	120.46 (17)
C8—C7—H7B	108.1	C17—C18—H18	119.8
C6—C7—H7B	108.1	C13—C18—H18	119.8
H7A—C7—H7B	107.3	C32—C31—C36	118.56 (15)
C9—C8—C7	116.28 (16)	C32—C31—C3	121.06 (14)
C9—C8—H8A	108.2	C36—C31—C3	120.38 (14)
C7—C8—H8A	108.2	C31—C32—C33	120.92 (15)
C9—C8—H8B	108.2	C31—C32—H32	119.5
C7—C8—H8B	108.2	C33—C32—H32	119.5
H8A—C8—H8B	107.4	C34—C33—C32	119.21 (16)
C8—C9—C10	115.62 (18)	C34—C33—H33	120.4
C8—C9—H9A	108.4	C32—C33—H33	120.4
C10—C9—H9A	108.4	C33—C34—C35	121.11 (15)
C8—C9—H9B	108.4	C33—C34—Cl1	119.88 (13)
C10—C9—H9B	108.4	C35—C34—Cl1	119.00 (13)
H9A—C9—H9B	107.4	C34—C35—C36	119.39 (16)
C9—C10—C11	115.86 (15)	C34—C35—H35	120.3
C9—C10—H10A	108.3	C36—C35—H35	120.3
C11—C10—H10A	108.3	C31—C36—C35	120.79 (16)
C9—C10—H10B	108.3	C31—C36—H36	119.6
C11—C10—H10B	108.3	C35—C36—H36	119.6
H10A—C10—H10B	107.4	N2—C38—C2	176.07 (19)
C4—C11—C10	112.58 (15)	C1—N1—H1	107.2 (15)
C4—C11—H11A	109.1	C5—N3—C1	123.00 (13)
C10—C11—H11A	109.1	C5—N3—C12	120.87 (13)
C4—C11—H11B	109.1	C1—N3—C12	115.95 (13)
			
N1—C1—C2—C3	178.97 (16)	C15—C16—C17—C18	0.5 (3)
N3—C1—C2—C3	−0.5 (2)	C16—C17—C18—C13	0.5 (3)
N1—C1—C2—C38	−1.8 (2)	C14—C13—C18—C17	−0.9 (3)
N3—C1—C2—C38	178.71 (13)	C12—C13—C18—C17	176.46 (16)
C38—C2—C3—C4	−178.72 (14)	C2—C3—C31—C32	−79.6 (2)
C1—C2—C3—C4	0.4 (2)	C4—C3—C31—C32	100.90 (19)
C38—C2—C3—C31	1.8 (2)	C2—C3—C31—C36	100.99 (19)
C1—C2—C3—C31	−179.06 (13)	C4—C3—C31—C36	−78.5 (2)
C2—C3—C4—C5	−0.5 (2)	C36—C31—C32—C33	1.4 (3)
C31—C3—C4—C5	179.02 (14)	C3—C31—C32—C33	−177.97 (15)
C2—C3—C4—C11	172.41 (14)	C31—C32—C33—C34	−1.1 (3)
C31—C3—C4—C11	−8.1 (2)	C32—C33—C34—C35	−0.1 (3)
C3—C4—C5—N3	0.6 (2)	C32—C33—C34—Cl1	178.95 (13)
C11—C4—C5—N3	−172.17 (14)	C33—C34—C35—C36	0.9 (3)
C3—C4—C5—C6	−179.76 (14)	Cl1—C34—C35—C36	−178.19 (14)
C11—C4—C5—C6	7.5 (2)	C32—C31—C36—C35	−0.7 (3)
C4—C5—C6—C7	−88.02 (19)	C3—C31—C36—C35	178.75 (16)
N3—C5—C6—C7	91.64 (18)	C34—C35—C36—C31	−0.5 (3)
C5—C6—C7—C8	74.9 (2)	C3—C2—C38—N2	−174 (3)
C6—C7—C8—C9	−67.4 (3)	C1—C2—C38—N2	7 (3)
C7—C8—C9—C10	99.1 (2)	C4—C5—N3—C1	−0.7 (2)
C8—C9—C10—C11	−55.1 (2)	C6—C5—N3—C1	179.62 (13)
C5—C4—C11—C10	88.34 (19)	C4—C5—N3—C12	174.19 (14)
C3—C4—C11—C10	−84.35 (19)	C6—C5—N3—C12	−5.5 (2)
C9—C10—C11—C4	−52.2 (2)	N1—C1—N3—C5	−178.89 (14)
N3—C12—C13—C14	−132.69 (16)	C2—C1—N3—C5	0.6 (2)
N3—C12—C13—C18	49.9 (2)	N1—C1—N3—C12	6.0 (2)
C18—C13—C14—C15	0.4 (3)	C2—C1—N3—C12	−174.52 (12)
C12—C13—C14—C15	−177.01 (17)	C13—C12—N3—C5	86.24 (17)
C13—C14—C15—C16	0.5 (3)	C13—C12—N3—C1	−98.50 (16)
C14—C15—C16—C17	−1.0 (3)		

### Hydrogen-bond geometry (Å, º)

Cg1 is the centroid of the phenyl ring.

**Table:**

*D*—H···*A*	*D*—H	H···*A*	*D*···*A*	*D*—H···*A*
C32—H32···N1^i^	0.93	2.55	3.423 (2)	156
C11—H11*B*···*Cg*1^ii^	0.97	2.91	3.5642 (2)	126

Symmetry codes: (i) −*x*+1, −*y*+2, −*z*+1; (ii) −*x*, −*y*+1, −*z*+1.
